# Successful retreatment with sofosbuvir plus ledipasvir for cirrhotic patients with hepatitis C virus genotype 1b, who discontinued the prior treatment with asunaprevir plus daclatasvir: A case series and review of the literature

**DOI:** 10.18632/oncotarget.23768

**Published:** 2017-12-29

**Authors:** Yuki Haga, Tatsuo Kanda, Shin Yasui, Masato Nakamura, Yoshihiko Ooka, Koji Takahashi, Shuang Wu, Shingo Nakamoto, Makoto Arai, Tetsuhiro Chiba, Hitoshi Maruyama, Osamu Yokosuka, Nobuo Takada, Mitsuhiko Moriyama, Fumio Imazeki, Naoya Kato

**Affiliations:** ^1^ Department of Gastroenterology, Chiba University, Graduate School of Medicine, Inohana, Chuo-ku, Chiba, Japan; ^2^ Division of Gastroenterology and Hepatology, Department of Internal Medicine, Nihon University School of Medicine, Itabashi-ku, Tokyo, Japan; ^3^ Department of Molecular Virology, Chiba University, Graduate School of Medicine, Inohana, Chuo-ku, Chiba, Japan; ^4^ Department of Internal Medicine, Toho University Sakura Medical Center, Shimoshizu, Sakura, Japan; ^5^ Safety and Health Organization, Chiba University, Yayoicho, Inage-ku, Chiba, Japan

**Keywords:** hepatitis C virus, direct-acting antiviral failure, non-structural 5A (NS5A) inhibitors, resistance-associated variants, case reports

## Abstract

**Background:**

Interferon-free treatment results in higher sustained virologic response (SVR) rates, with no serious adverse events in hepatitis C virus (HCV)-infected patients. However, in some patients with treatment-failure in HCV NS5A inhibitor-including interferon-free regimens, the treatment-emergent HCV NS5A resistance-associated variants (RAVs), which are resistant to interferon-free retreatment including HCV NS5A inhibitors, are observed. In HCV-infected Japanese patients with daclatasvir and asunaprevir treatment failure, retreatment with sofosbuvir and ledipasvir could lead to only ∼70% SVR rates.

**Case summary:**

Three HCV genotype (GT)-1b-infected cirrhotic patients who discontinued the combination of daclatasvir and asunaprevir due to adverse drug reactions within 4 weeks; retreatment with sofosbuvir and ledipasvir combination could result in SVR in these patients without RAVs. One HCV GT-1b-infected cirrhotic patient who discontinued the combination of daclatasvir and asunaprevir due to viral breakthrough at week 10; retreatment with sofosbuvir and ledipasvir combination for this patient with the treatment-emergent HCV NS5A RAV-Y93H resulted in viral relapse at week 4 after the end of the treatment.

**Conclusion:**

Retreatment with sofosbuvir and ledipasvir is effective for HCV GT-1b patients who discontinue the combination of daclatasvir and asunaprevir within 4 weeks. The treatment response should be related to the existence of treatment-emergent HCV NS5A RAVs, but may not be related to the short duration of treatment.

## INTRODUCTION

Chronic hepatitis C virus (HCV) infection remains one of the most serious health problems worldwide because it frequently leads to liver cirrhosis and hepatocellular carcinoma (HCC) [1, 2]. Sustained virologic response (SVR) could lead to biochemical normalization and histopathological improvement in patients with HCV infection [2]. Recent advances in the development of direct-acting antiviral agents (DAAs) have enabled us to eradicate HCV more effectively, even in patients who are intolerant to interferon (IFN)-including treatment or who are non-responders to interferon-including treatment. Interferon-free combination treatment with HCV NS5A inhibitor daclatasvir and HCV NS3 protease inhibitor asunaprevir was safe and showed a few adverse events in HCV genotype (GT)-1b-infected patients [3–6]. It has also been reported that the existence of resistance-associated variants (RAVs), such as NS5A-Y93H polymorphism, is associated with virologic failure in daclatasvir and asunaprevir combination treatment [6, 7].

Sofosbuvir is an oral nucleotide HCV NS5B polymerase inhibitor, and ledipasvir is an HCV NS5A inhibitor. Interferon-free combination treatment with these drugs has resulted in high rates of SVR among patients with HCV GT-1, even among with patients with cirrhosis [8–10]. It is unknown whether retreatment with sofosbuvir and ledipasvir are effective for HCV GT-1 patients who undergo prior treatment with HCV NS5A inhibitors [11, 12]. In Japanese DAA-naïve patients with HCV infection, sofosbuvir and ledipasvir can lead to 99% SVR rates [10]. However, in HCV-infected Japanese patients with daclatasvir and asunaprevir treatment failure, retreatment with sofosbuvir and ledipasvir may lead to only ∼70% SVR rates [11, 12]. Here, we report on four cirrhotic patients who discontinued daclatasvir and asunaprevir treatment, with adverse events, and completed retreatment with sofosbuvir plus ledipasvir for 12 weeks. Three patients, who discontinued this combination within 4 weeks, achieved SVR by retreatment with sofosbuvir plus ledipasvir for 12 weeks; but one patient who discontinued this combination at week 10, did not achieve SVR by retreatment with sofosbuvir plus ledipasvir for 12 weeks. The treatment response should be related to the existence of treatment-emergent HCV NS5A RAVs, but may not be related to the short duration of treatment.

## CASE 1

A 58-year-old woman was diagnosed with HCV GT-1b infection in another hospital. She underwent appendectomy at age 15. She was not a drinker but had a medical history of hypothyroidism and took levothyroxine.

She was previously treated with interferon therapy at the previous hospital, but she was non-responsive. She was treated with daclatasvir (60 mg daily) and asunaprevir (200 mg daily) in 2014. However, this treatment was discontinued at 4 weeks because of polymorphism exudative erythema, and she required temporal steroid therapy. She was referred to our hospital in 2016 to be retreated with sofosbuvir (400 mg daily) and ledipasvir (90 mg daily). Laboratory data at the start of retreatment are shown in Table 1. Although liver biopsy was not performed, her liver stiffness and low platelet counts indicated cirrhosis. HCV NS5A-L31 and -Y93 sequencing using a real-time polymerase chain reaction (PCR) system and a cycling probe assay [13] revealed no evidence of HCV NS5A RAVs. She received full doses of both sofosbuvir and ledipasvir for 12 weeks, and no adverse events were observed. The estimated glomerular filtration rate (eGFR) did not change during the therapy. Serum HCV RNA was not detected 4 weeks after commencing treatment, and it remained undetectable until the end of the treatment. The patient achieved SVR at 12 weeks after the end of treatment (SVR12).

**Table 1:** Baseline laboratory data before the retreatment with sofosbuvir and ledipasvir in the present study

	Case 1	Case 2	Case 3	Case 4
Age (years)	58	68	78	78
Gender	Female	Female	Male	Female
Duration of prior treatment with daclatasvir and asunaprevir (weeks)	4	3	2	10
Adverse events in prior treatment	Polymorphism exudative erythema	Fever	Cough and nasal mucus	Viral breakthrough
Body length (cm)	157	155	167	155
Body weight (kg)	49	50	57.8	52
Body mass index (kg/m^2^)	19.9	20.8	20.8	21.6
White blood cell count (/μL)	3700	3400	5400	4100
Red blood cell count (10^4^/μL)	384	468	468	414
Hemoglobin (g/dL)	12.9	13.9	11.7	12.2
Platelet counts (10^3^/μL)	82	143	161	12.5
Prothrombin time (%)	77	123	102	80
Total bilirubin (mg/dL)	1.2	0.6	0.5	0.8
Aspartate aminotransferase (IU/L)	62	23	56	56
Alanine aminotransferase (IU/L)	68	22	44	52
Lactate dehydrogenase (IU/L)	235	173	214	208
Alkaline phosphatase (IU/L)	222	222	299	476
γ-glutamyl transpeptidase (IU/L)	52	35	34	40
Total protein (g/dL)	7.0	7.1	7.6	7.1
Albumin (g/dL)	3.3	3.8	3.6	3.5
Blood urea nitrogen (mg/dL)	13	16	18	16
Creatinine (mg/dL)	0.60	0.74	0.82	0.62
Estimated glomerular filtration rates (ml/min/1.73 m^2^)	78.2	59.4	68.8	69.0
α-fetoprotein (ng/mL)	88.2	4.2	7.2	11.3
Child-Pugh classification	A	A	A	A
Liver stiffness (kPa)	24.8	unknown	16.5	31.2
HCV RNA (logIU/mL)	4.7	6.3	4.7	6.6
HCV genotype	1b	1b	1b	1b
Previous interferon treatment	Yes	Yes	Yes	Yes
IL28B rs8099917	unknown	unknown	TT	unknown
NS5A RAVs at L31/Y93	none	none	none	Y93H (>99%)

RAVs, resistance-associated variants

## CASE 2

A 68-year-old woman was diagnosed with HCV GT-1b infection at another hospital. She received a blood transfusion when she gave birth and denied other risk factors for HCV infection, including tattoos or intravenous drug use. She was not a drinker but she had a medical history of hypertension and benign paroxysmal positional vertigo and took several medications for these diseases.

She was previously treated with peginterferon-α and ribavirin in 2010 but the treatment was discontinued because of faintness. Next, she was treated with daclatasvir and asunaprevir in 2015. However, this interferon-free treatment was discontinued at 3 weeks because of fever. She was referred to our hospital in 2016 for retreatment with sofosbuvir and ledipasvir. Laboratory data before the retreatment are shown in Table 1. The patient did not undergo liver biopsy, but she was diagnosed with cirrhosis because of US imaging and low platelet counts. Direct sequencing of HCV NS5A-L31 and NS5A-Y93 revealed no evidence of HCV NS5A RAVs. She received full doses of sofosbuvir (400 mg daily) and ledipasvir (90 mg daily) for 12 weeks, and no adverse events were observed. The eGFR did not change during the treatment. Serum HCV RNA was not detected 4 weeks after commencing treatment and remained undetectable until the end of the treatment. She achieved SVR12.

## CASE 3

A 78-year-old man was diagnosed with HCV GT-1b infection in 1995. He received a distal gastrectomy for duodenum ulcer and denied other risk factors for HCV infection, including tattoos or intravenous drug use. The patient was a social drinker, had a medical history of pulmonary emphysema, and took several medications for this disease.

He was treated with 180 μg of peginterferon-α-2a in 2005. However, the therapy was discontinued because he was diagnosed with drug-induced interstitial pneumonia. From 2011 to 2014, the patient was diagnosed with hepatocellular carcinoma (HCC) and treated once with transarterial chemoembolisation (TACE) and five times radiofrequency ablation (RFA). At the end of 2014, enhanced abdominal computed tomography (CT) findings showed no HCCs in the liver and no ascites, and the patient was treated with 60 mg of daclatasvir and 200 mg of asunaprevir at the beginning of 2015. However, this treatment was discontinued at 2 weeks because of cough and nasal mucus [6]. These symptoms disappeared immediately after stopping this medication.

The patient wanted to be retreated for his chronic hepatitis C; thus, he was retreated with sofosbuvir (400 mg daily) plus ledipasvir (90 mg daily) at the end of 2015. Laboratory data before retreatment are shown in Table 1. He was diagnosed with cirrhosis because of liver stiffness. HCV NS5A-L31 and NS5A-Y93 sequencing using a real-time PCR system and cycling probe assay [13] revealed no evidence of HCV NS5A RAVs. He received the full doses of sofosbuvir and ledipasvir for 12 weeks and no adverse events were observed. The eGFR did not change during the therapy. Serum HCV RNA was not detected at week 4 after the commencement of treatment, and it remained undetectable until the end of the treatment. He achieved SVR12 [10].

## CASE 4

A 78-year-old woman has been diagnosed with HCV GT-1b infection since 2002. She received cholecystectomy and blood transfusion at 1962, and denied other risk factors for HCV infection, including tattoos or intravenous drug use. The patient had a medical history of hyperthyroidism but was not a drinker. He was treated with peginterferon-α-2a with or without ribavirin from 2005-2008. However, she was null responder for interferon-including regimens.

In the previous hospital, she was administered 60 mg of daclatasvir and 200 mg of asunaprevir at April, 2015. However, this treatment was discontinued at 10 weeks because of viral breakthrough.

She wanted to be retreated for chronic hepatitis C and was referred to our hospital. Laboratory data before treatment are shown in Table 1. She was diagnosed with cirrhosis because of liver stiffness and low platelet counts. HCV NS5A-L31 and NS5A-Y93 sequencing using a real-time PCR system and cycling probe assay [13] revealed that she had a HCV NS5A RAV-Y93H. As no HCV NS5A RAVs were detected before the combination treatment of daclatasvir and asunaprevir at the previous hospital, we considered that this HCV NS5A RAV-Y93H was emerged by this combination treatment.

She was retreated with sofosbuvir (400 mg daily) plus ledipasvir (90 mg daily) from 2016-2017. No adverse events were observed, and she received the full doses of sofosbuvir and ledipasvir for 12 weeks. The eGFR did not change during the therapy. Serum HCV RNA was not detected at 4 weeks after the commencement of treatment, and it remained undetectable until the end of the treatment. However, HCV RNA was relapsed at week 4 after the end of the treatment.

Previously, we reported that daclatasvir and asunaprevir combination treatment could lead to 94.4% SVR12 in HCV GT-1b-infected Japanese patients after screening for HCV NS5A RAVs [6]. However, some patients discontinued this combination therapy due to adverse events and remained HCV RNA positive. The ideal retreatment for patients who failed the combination of daclatasvir and asunaprevir has not been established.

In the American Association for the Study of the Liver Diseases (AASLD) guidelines, testing for HCV NS5A RAVs is recommended for the NS5A inhibitor, including daclatasvir treatment-experienced patients [14]. The guidelines from the Asian Pacific Association for the Study of the Liver (APASL) also recommend that RAVs with target regions should be examined before starting any treatments in patients who undergone HCV NS5A inhibitor-including regimens [15].

The treatment with sofosbuvir and ledipasvir achieved high rates of SVR with good safety profiles in HCV NS5A inhibitors-naïve patients [8–10]. However, it has been reported that HCV NS5A RAVs undermine the virologic effects of combination treatment of daclatasvir and asunaprevir in Japanese patients [5, 6]. In three patients (cases 1-3), no RAVs were observed in the NS5A-L31/Y93 regions and SVR was achieved by the retreatment with sofosbuvir and ledipasvir.

We reported that three HCV GT-1b-infected cirrhotic patients who discontinued the combination of daclatasvir and asunaprevir due to adverse drug reactions within 4 weeks and that the retreatment with sofosbuvir and ledipasvir combination could result in SVR in these three patients. This retreatment had no serious adverse events. The treatment response might be related to the existence of treatment-emergent HCV NS5A RAVs, but may not be related to the short duration of treatment. It was reported that all treatment-failure patients without HCV NS5A RAVs underwent previous treatment, including HCV NS5A inhibitors, for 8 weeks or less and that patients with HCV NS5A RAVs underwent previous treatment, including HCV NS5A inhibitors, for over 8 weeks [16].

One patient (case 4) discontinued the combination of daclatasvir and asunaprevir due to viral breakthrough at week 10. She had a treatment-emergent HCV NS5A RAV-Y93H. She did not achieve SVR by the combination retreatment of sofosbuvir and ledipasvir for 12 weeks.

## CONCLUSIONS

Retreatment with sofosbuvir and ledipasvir successfully eradicated HCV RNA in three patients without any serious adverse events. This combination might be an effective option for HCV GT-1b patients without treatment-emergent HCV NS5A RAVs, who discontinue the combination of daclatasvir and asunaprevir within 4 weeks.
